# Effect of prostaglandin E_2 _injection on the structural properties of the rat patellar tendon

**DOI:** 10.1186/1758-2555-4-2

**Published:** 2012-01-09

**Authors:** Scott T Ferry, Hessam M Afshari, Justin A Lee, Laurence E Dahners, Paul S Weinhold

**Affiliations:** 1 Department of Orthopaedics, University of North Carolina at Chapel Hill, CB# 7055, Chapel Hill, NC 27599-7055 USA

## Abstract

**Background:**

Increased tendon production of the inflammatory mediator prostaglandin E_2 _(PGE_2_) has been suggested to be a potential etiologic agent in the development of tendinopathy. Repeated injection of PGE_2 _into tendon has been proposed as a potential animal model for studying treatments for tendinopathy. In contrast, nonsteroidal anti-inflammatory drugs (NSAIDs) which inhibit PGE_2 _production and are commonly prescribed in treating tendinopathy have been shown to impair the healing of tendon after acute injury in animal models. The contradictory literature suggests the need to better define the functional effects of PGE_2 _on tendon. Our objective was to characterize the effects of PGE_2 _injection on the biomechanical and biochemical properties of tendon and the activity of the animals. Our hypothesis was that weekly PGE_2 _injection to the rat patellar tendon would lead to inferior biomechanical properties.

**Methods:**

Forty rats were divided equally into four groups. Three groups were followed for 4 weeks with the following peritendinous injection procedures: No injection (control), 4 weekly injections of saline (saline), 4 weekly injections of 800 ng PGE_2 _(PGE_2_-4 wks). The fourth group received 4 weekly injections of 800 ng PGE_2 _initially and was followed for a total of 8 weeks. All animals were injected bilaterally. The main outcome measurements included: the structural and material properties of the patellar tendon under tensile loading to failure, tendon collagen content, and weekly animal activity scores.

**Results:**

The ultimate load of PGE_2_-4 wks tendons at 4 weeks was significantly greater than control or saline group tendons. The stiffness and elastic modulus of the PGE_2 _injected tendons at 8 weeks was significantly greater than the control or saline tendons. No differences in animal activity, collagen content, or mean fibril diameter were observed between groups.

**Conclusions:**

Four weekly peritendinous injections of PGE_2 _to the rat patellar tendon were not found to be an effective model of clinical tendinopathy. In contrast, improved structural and material properties of the patellar tendon were found after PGE_2 _injection. While PGE_2 _has been thought to have a contributory role in the development of tendinopathy and anti-inflammatory medications remain a common treatment, our results suggest a positive role of PGE_2 _in tendon remodeling in some circumstances.

## Background

Tendinopathy is a frequent source of pain and disability seen in clinical practice. Common sites of tendinopathy include the rotator cuff, the common extensor origin at the elbow, the patellar tendon, and the Achilles tendon. Histologically, tendinopathy is characterized by degeneration and disorganization of collagen fibrils, increased mucoid ground substance, and the notable absence of inflammatory cells [1]. In the clinical setting, tendinopathy ranges from activity related pain to frank rupture. The pathophysiology behind development of this condition as well as the optimum treatment remains controversial. An ideal animal model of tendinopathy would produce similar histologic findings, would decrease biomechanical strength of the tendon, could be readily applied to different anatomic areas, and would share a common pathophysiology to human tendinopathies.

Several theories exist about the pathophysiology behind the development of tendinopathy, but most involve some type of cellular response induced by repetitive motions [2,3]. One theory involves the local release of inflammatory mediators by fibroblasts in response to mechanical loading. Cyclical loading of tendon fibroblasts *in vitro *has been shown to increase local concentrations of PGE_2 _and leukotriene B_4 _[2,3]. Cyclical loading has also been shown to increase PGE_2 _and nitric oxide levels in a tendon explant system [4]. PGE_2 _has been implicated as a potential etiologic agent in the development of tendinopathy and as a possible inducer of degradative enzyme activity [5].

In attempts to determine how local prostaglandin levels may affect tendon *in vivo*, a few studies have investigated local injection of prostaglandin E_1 _(PGE_1_) or PGE_2 _as an animal model of tendinopathy. Sullo et al. injected the rat Achilles tendon with PGE_1 _and found increased cross sectional area and degenerative changes similar to human tendinopathy after 5 weeks [6]. Khan et al. injected PGE_2 _into rabbit patellar tendons and found loss of collagen fiber organization and decreased collagen fibril diameter compared to controls [7]. However, neither of these studies evaluated the effect of prostaglandin injection on the tendon biomechanical properties which are more functionally relevant to the risk of the tendon degenerating to a state for which the chance of rupture is elevated.

In contrast to the proposed role of prostaglandins in tendon degeneration, there exists alternative evidence that suggests a positive role of prostaglandins in tendon healing. Several acute tendon injury models have demonstrated that administration of cyclooxgenase inhibitors which act to decrease the production of prostaglandins during the healing period impair the biomechanical properties of the healing tissue [8,9]. Furthermore, it has been suggested nonsteroidal anti-inflammatory drugs (NSAIDs) may negatively influence tendon healing in tendinopathy and may contribute to the failed healing response [10]. Recent work has also shown that blockade of PGE_2 _production by NSAID intake can abolish exercise-induced increases in collagen synthesis in the human patellar tendon [11]. Past studies have found local administration of PGE_2 _in muscle in rabbits to stimulate fibrous tissue formation suggesting anabolic effects [12].

The contradictory literature concerning the positive or negative role of prostaglandins on tendon suggests the need for studies to better define the functional biomechanical effects of local application of PGE_2 _on tendon. Our objective was to characterize the effects of PGE_2 _injection on the biomechanical and biochemical properties of tendon and the activity of the animals. Our hypothesis was that weekly PGE_2 _injection to the rat patellar tendon would lead to inferior biomechanical properties.

## Methods

### Animal care and procedure

Protocols were approved by the University Institutional Animal Care and Use Committee. A total of 40 retired-breeder female Sprague-Dawley rats (350-500 g) were obtained from a commercial breeder. Four different study groups of ten animals each were used. A control group underwent no injections. The remaining groups underwent weekly peritendinous injections into both patellar tendons with a 27 gauge needle and a 100 microliter syringe. The saline group received injection of 50 microliters of 0.9% sodium chloride. The PGE_2_-4 wks and PGE_2_-8 wks groups had injections of 800 ng of PGE_2 _(P5640, Sigma-Aldrich, St. Louis, MO) in 50 microliters of 0.9% sodium chloride. Injections were performed at 0,7,14, and 21 days. Animals were anesthetized with isoflurane and the knees were shaved using electric clippers. The skin was prepped with an alcohol solution and the knee was flexed to 90 degrees to tension the tendon. The tendon was then injected parallel thru its length to a point midway between the patella and tibial insertions [7]. The India ink of practice injections in cadavers was found to distribute superficially from the tendon, but stay beneath the peritenon. Thus, while the injection was intratendinous it may better be described as peritendinous. Animals were returned to their cages and allowed food, water, and activity ad libitum. All groups were sacrificed at 28 days, except the PGE_2_-8 wks group which was sacrificed at 56 days.

### Activity Monitoring

In order to determine if there was some disability related to the injections or PGE_2_, we monitored animal activity relative to pre-injection levels. This was completed for 6 animals in the saline group and 6 animals in the PGE_2_-8 wks group. We used a photoelectric sensors system to monitor animal activity [9]. The rats were housed in individual cages with the water source on one end and food on the other end. The photoelectric sensor (Q14, Banner Engineering, Minneapolis, MN) was set-up to bisect the mid-portion of the cage. The sensors were linked to a control module (Logo!, Siemens, New York, NY) that recorded a count each time the beam was crossed. One count was recorded each time the animal stepped into and subsequently out of the beam with a one-second delay. Total counts were recorded at daily intervals for the duration of the study. The seven daily counts across a week's interval were averaged to compute an average daily count for each week. The pre-injection daily count measurement was taken as the average of the daily activity counts during the 5 days pre-injection. The average daily count was used in the statistical analysis.

### Specimen Preparation

All animals were sacrificed by CO_2 _overdose and both hind limbs harvested. Half of the right specimens (N = 5 per group) were transferred to 10% neutral buffered formalin, sequentially decalcified, dehydrated, embedded in plastic, sectioned, stained with hematoxylin and eosin, and qualitatively examined for fiber arrangement and cellularity. The remaining right limb specimens were fixed for fibril diameter analysis. The left hind limb were placed in saline-soaked gauze and stored at -20°C until the time of mechanical testing. On the day of biomechanical testing, the quadriceps, patella, patellar tendon, and tibia were isolated and dissected free of other soft tissues.

### Mechanical Testing

The length of the tendon as viewed from the anterior side was measured using a digital micrometer. The cross sectional area of the tendon at its midpoint was measured using an area micrometer after 0.12 MPa of compression was applied for 2 minutes. Measurements were taken in triplicate and averaged. Spray irrigation with saline was used to keep the tissue moist during all procedures. Following dissection and measurement, the tibia was secured in a custom grip on a servohydraulic testing machine (8500; Instron, Norwood MA). The quadriceps muscle and tendon were secured in a custom designed cryogrip and the visible freeze line edge of the tissue was allowed to migrate to the superior edge of the patella. The tendon was preloaded to 0.5 N and then the complex was loaded to failure at a constant deformation rate of 0.08 mm/sec corresponding to a 1% strain rate per second. The load-deformation data was acquired via an analog to digital converter linked to a personal computer and the failure site was recorded. The structural properties of maximum load, linear stiffness between 25-75% load limits, energy absorbed, and deformation at ultimate load were determined as well as the corresponding material properties.

### Biochemical Analysis

Following the biomechanical testing, the patellar tendon was harvested and stored at -20°C. At the time of evaluation, the tissue was thawed and then dehydrated. Dry weight was recorded and the tissue was then subjected to papain digestion (0.5 mg/ml, Sigma-Aldrich, St. Louis, MO) in a 0.1 M phosphate buffer (pH 7) with 10 mM Cystine HCL & 2 mM EDTA at 60°C for 6 hours. The digest was used for the determination of collagen content. Collagen content of the digest was measured by hydroxyproline concentration by the method of Bergman & Loxley with a plate reader [13]. Standard curves were created using known concentrations of hydroxyproline (Sigma-Aldrich, St. Louis, MO).

### Fibril Diameter Analysis

Tendons for this analysis (n = 5 per group for the control and PGE2-8 wks groups) were fixed in 2% paraformaldehyde/2.5% glutaraldehyde in 0.15 M sodium phosphate buffer, pH 7.4, for 24 hours followed by post-fixation in 1% osmium tetroxide in 0.15 M sodium phosphate buffer, pH 7.4, for 1 hour. Samples were dehydrated in a graded series of ethanols, followed by propylene oxide, and infiltrated and embedded in Polybed 812 resin (Polysciences, Inc., Warrington, PA). Tendon cross sections of 70 nm thickness were stained with 6% methanolic uranyl acetate and Reynolds' lead citrate. Sections were observed using a LEO EM-910 transmission electron microscope operating at 80 kV (Carl Zeiss SMT, Peabody, MA) and images were taken using a Gatan Orius SC1000 CCD camera with Digital Micrograph 3.11.0 (Gatan, Inc., Pleasanton, CA). Two regions of interest of (approximate size of 1.26 μm × 1.05 μm) from each of three images (approximate size of 5.41 μm × 3.84 μm) of each animal were used to compute the average fibril diameter. Fibril diameters were measured for all fibrils in the region of interest using a custom script written in a commercially available image analysis program (Vision 6.0, National Instruments Inc., Austin, TX).

### Statistical Analysis

Biomechanical parameters, collagen content, and collagen fibril diameters were evaluated with a one way ANOVA, followed by Holm-Sidak multiple comparison testing. Activity counts from preinjection to 8 weeks were analyzed using a two-way repeated measures ANOVA for the factors of time (repeated factor) and group.

## Results

Forty animals were included in the study. One animal was excluded from biomechanical testing in the PGE_2_-8 wks groups due to tissue damage during dissection and five specimens were excluded because the tensile failure occurred at the quadriceps insertion of the patella during biomechanical testing. The remaining specimens failed at the patellar tendon insertions at either the tibial tubercle (T) or patella (P). The failure site distribution for each group was as follows: Control (6P, 1T), Saline (7P, 2T), PGE2-4wks (7P, 2T), PGE2-8 wks (6P, 3T).

**Figure 1:**
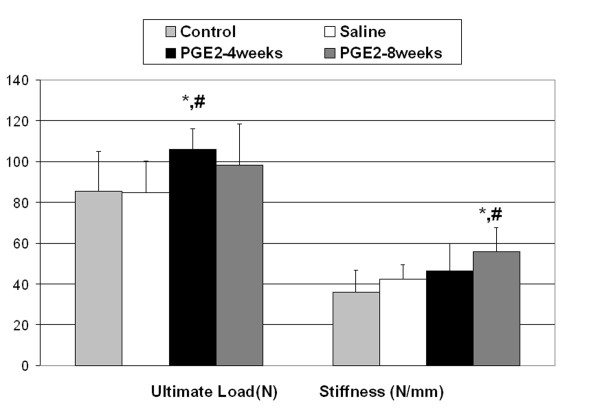
**Ultimate tensile load and stiffness of the rat patellar tendon was increased with PGE_2 _injection**. * Significant difference vs. control.(P < 0.05) # Significant difference vs. saline injected group. (P < 0.05).

The ultimate load of the tendons in the PGE_2_-4 wks were significantly greater than the control and the sodium chloride injected tendons (Figure 1) (P < 0.05). The ultimate tensile stress was not significantly different between groups though the PGE_2 _groups showed a trend to be stronger than the sodium chloride injected group (Table 1). The PGE_2_-8 wks group tendons had significantly greater structural stiffness than the control and the sodium chloride injected tendons (Figure 1) (P < 0.05). The PGE_2_-8 wks group tendons also displayed a significantly greater elastic modulus than the control and the sodium chloride injected tendons (Table 1). In addition, the PGE_2_-8 wks group tendons displayed significantly less deformation prior to the ultimate load compared to the control tendons (Table 2 P < 0.05) and a trend for a similar effect for the strain at ultimate load (Table 1). Finally, a significant increase in the cross sectional area of the tendons in the sodium chloride injected and PGE_2_-4 wks tendon as compared to the control tendons (P < 0.05) (Table 2) was found. There were no significant differences between groups with respect to length, energy absorbed or energy density (Tables 1 &2). There were no differences between initial or final body weights for animals between groups.

**Table 1:** Material properties of the rat patellar tendon for the 4 groups (Mean ± SD)

Group	Ultimate Stress (MPa)	Strain to Ultimate Load (mm/mm)	Elastic Modulus (MPa)	Energy Density to Ultimate Load (mJ/mm^3^)
Control	26.2 ± 6.8	0.34 ± 0.08	84.5 ± 23.2	4.66 ± 2.38
Saline	21.6 ± 4.7	0.28 ± 0.06	83.2 ± 17.1	3.14 + 0.98
PGE2-4 wks	27.1 ± 5.3	0.31 ± 0.09	90.0 ± 18.2	4.47 + 1.67
PGE2-8 wks	28.2 ± 5.9	0.24 ± 0.05	124.0 ± 35.0*,^#^,^+^	3.96 ± 1.22
F-test	P = 0.091	P = 0.053	P = 0.004	P = 0.226

* Indicates difference from control group (P < 0.05) # Indicates difference from saline-injected group (P < 0.05).+ Indicates difference from PGE2-4 wks injection group (P < 0.05).

**Table 2:** Structural properties of the rat patellar tendon for the 4 groups (Mean ± SD)

Group	Energy to Ultimate Load (J)	Displacement at Ultimate Load (mm)	Length (mm)	Cross-sectional area (mm^2^)
Control	0.116 ± 0.053	2.64 ± 0.65	7.72 ± 0.57	3.30 ± 0.43
Saline	0.096 ± 0.028	2.18 ± 0.44	7.70 ± 0.25	3.98 ± 0.50*
PGE2-4 wks	0.132 ± 0.032	2.42 ± 0.65	7.81 ± 0.30	4.02 ± 0.75*
PGE2-8 wks	0.105 ± 0.029	1.87 ± 0.31*	7.66 ± 0.26	3.49 ± 0.28
F-test	P = 0.187	P = 0.036	P = 0.83	P = 0.021

* Indicates difference from control group (P < 0.05)

**Figure 2:**
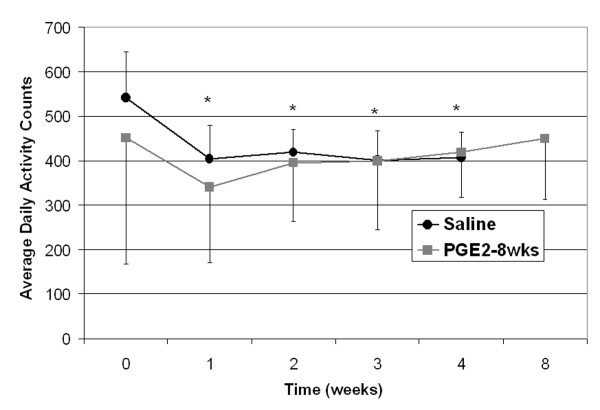
**Average daily activity counts across weekly intervals for the animals**. Counts at week zero were collected before injections were started. Injections stopped after 3 weeks in the PGE2-8 wk group. * Significant difference from week 0 (pre-injection) counts independent of group.(P < 0.05).

There was a significant decrease in average daily activity counts from the pre-injection counts for the post-injection times of 1, 2, 3, and 4 weeks independent of group (Figure 2) (P < 0.05). There was no significant difference in activity counts between the PGE_2 _and saline injected tendons. There was also no difference in activity counts in the PGE_2 _injected animals at 0 weeks compared to 8 weeks and at 4 weeks compared to 8 weeks.

**Figure 3:**
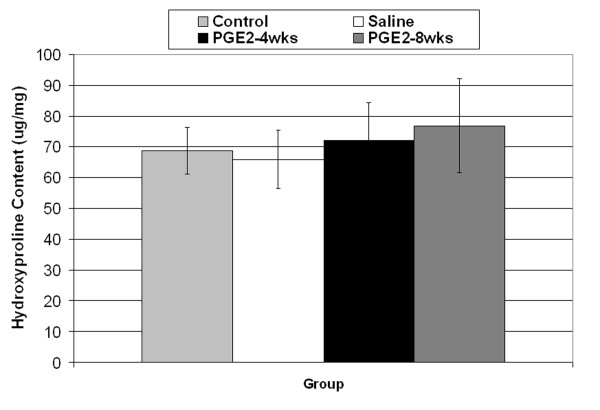
**Collagen content of the rat patellar tendon tissue did not differ among the 4 groups (P > 0.05)**.

**Figure 4:**
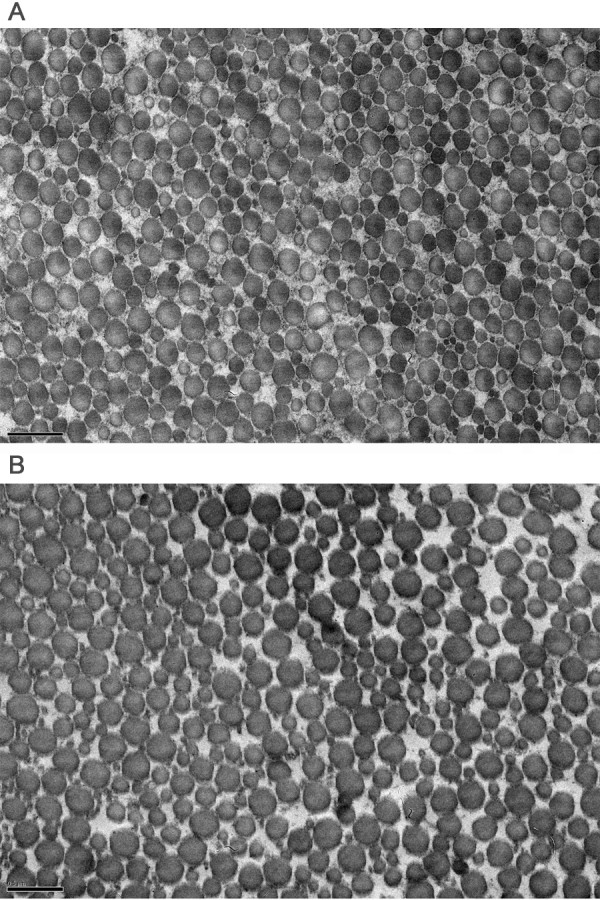
**Representative transmission electron microscopy images of the cross-section of the rat patellar tendon**. Image (A) is for a control rat and image (B) is for a PGE2-8 wk treated rat. Scale bar of the electron micrographs = 500 nm. The mean fibril diameter (Mean ± SD) of the rat patellar tendons (n = 5) was found to be similar (P > 0.05) in the control (154 ± 45) and PGE2-8 wks (167 ± 50) groups.

**Figure 5:**
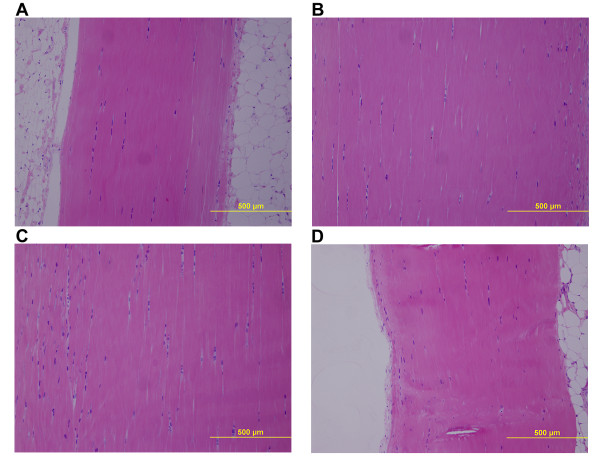
**Representative images of hematoxylin and eosin stained patellar tendon sections from the 4 groups**. The images show a lack of change in the matrix organization for the different groups: A) Control, B) PGE2-4 wks rat1, C) PGE2-4 wks rat2, D) PGE2-8 wks. (scale bar = 500 μm).

No difference in collagen content was found between the groups (Figure 3). The specimen numbers for the collagen content evaluations were Control(9), Saline(4), PGE2-4 wks(7), PGE2-and 8 wks (10). Fibril diameter analysis of the control and PGE_2_-8 wks groups revealed no difference in mean fibril diameter (Figure 4) between these groups. Histologic specimens demonstrated significant sectioning and orientation artifact that precluded a quantitative analysis. In the zones free of section artifact that were located more centrally or distally within the tendon it was qualitatively observed that there were no signs of collagen fiber disorganization or degeneration in the PGE_2 _injected tissue (Figure 5).

## Discussion

The results from our study did not support the hypothesis that the biomechanical properties would be inferior after PGE_2 _injection, rather, PGE_2 _appeared to produce the opposite effect. In our study the local application of PGE_2 _improved the structural strength and structural/material stiffness properties of the tendon. The increase in stiffness was sustained 4 weeks after the injections were stopped at 4 weeks suggesting these improvements were not transient. The increase in cross-sectional area of the PGE_2_-4 wks tendons suggests that this improvement in structural properties initially resulted from tendon hypertrophy. This hypertrophy corresponds with recent work that has shown that inhibition of PGE_2 _production *in vivo *can block exercise-induced increases in collagen synthesis of the patellar tendon in humans [11]. In addition, past work has shown local PGE_2 _administration to increase fibrous tissue formation in muscle in an *in vivo *rabbit model [12]. The improvement in stiffness in the PGE_2_-8 wks tendons of our study may have been due material changes as the elastic modulus was increased. However, material changes were not detected in the collagen content or mean fibril diameter evaluations.

There are several possible explanations for the difference in tissue response observed in this study compared to the hypothesized degenerative tissue response based on previous reports [6,7]. Our model may not have shown the degenerative effects if the dosage of PGE_2 _was inadequate, if the tendon was not exposed to PGE_2 _for a long enough time, or if the testing was completed at too early or late of a time point. The PGE_2 _injection model is likely an oversimplification of the complexity of clinical tendinopathy, and it is possible that administration of PGE_2 _in concert with exercise loading or additional inflammatory mediators that are upregulated with mechanical loading may lead to a tendon with degenerative properties. The dosage of PGE_2 _used in our study was similar to that used in a previous rabbit patellar tendon study observing a degenerative tissue response to PGE_2 _injection [6,7]. It is possible that with the larger volume of the rabbit patellar tendon compared to the rat, the injected PGE_2 _solution may have had a greater propensity to remain intratendinously causing a greater effect on the internal fibroblasts and collagen fibrils. In our study some of the injection solution was observed to leak from the tendon, but stay beneath the peritenon. Thus, the internal fibroblast may have been exposed to lower concentrations of PGE_2 _than desired. This may have contributed to the lack of change in fibril diameter and matrix organization observed in this study as compared to previous work [7]. If the collagen fibril changes to PGE_2 _injection reported in the previous rabbit patellar tendon model were a transient response, the slower rate of metabolism in the rabbit compared to the rat may have resulted in these changes dissipating prior to our examination in our rat model. The timing of our evaluation in our study was more extended than previous animal studies [6,7]. This might suggest that previously reported histological changes may not have been degenerative changes but simply transient effects in the tissue prior to obtaining its superior structural properties.

An alternative explanation for our results is that the PGE_2 _production with tendon loading actually plays a positive role in stimulating tendon remodeling and that PGE_2 _alone is not the primary component of the pathologic development of tendinopathy. In support of PGE_2_'s positive role in stimulating tendon remodeling, recent studies have shown that inhibitors of prostaglandin production, cyclooxgenase (COX) inhibitors, can impair the tensile strength of healing tendon tissue after transection injury [8,9]. In interpreting these COX inhibitor studies it is important to consider that these studies do not simply involve reduction of PGE_2_, but can also influence other elements of arachidonic acid metabolism such as the production of leukotreines and PGD*_2 _*[2,3,14]. Furthermore, the timing of the administration of COX inhibitors during tendon healing appears to be important as early administration appears to impair healing while delayed administration may improve the material properties of the tissue [15]. It is also important to note that a number of *in vitro *studies of human tendon fibroblasts from healthy tissue have demonstrated negative effects of exogenous PGE_2 _exposure on proliferation, collagen synthesis, and tendon stem cell differentiation [5,16]. In contrast, the findings of *in vitro *studies investigating the effect of COX inhibitors on tendon fibroblasts would suggest the opposite effect of PGE_2 _on proliferation as such inhibitors have been found to decrease cell proliferation [17,18]. There will always be some uncertainty regarding whether the mechanical and biochemical environment of the *in vitro *setting are representative of the *in vivo *setting of tendon healing. Recent work has shown that cultured tendon cells of intact tendon do not demonstrate the same cellular activity as healing tendon cells [19]. Furthermore, *in vivo *microdialysis studies examining the effect of COX inhibition on exercise-induced collagen synthesis would suggest that PGE_2 _increases collagen synthesis [11] as contrasted with *in vitro *work which suggests PGE2 decreases collagen synthesis [5]. Finally, past studies have shown that the injection of fatty acid preparations that are similar to the arachidonic acid precursors of prostaglandins can increase the strength of ligament and tendon [20,21]. Thus, it is apparent from these studies there still exists a great amount of uncertainty regarding the effects of PGE_2 _on tendon *in vivo*.

While there is little literature regarding the direct effect of prostaglandins on the biomechanical properties of tendon and ligament, there is a substantial body of evidence regarding the effects of prostaglandins on bone. Multiple *in vivo *studies have demonstrated an anabolic effect on bone with subcutaneous or systemic administration of prostaglandins. These studies have demonstrated increased bone formation in multiple sites with prostaglandin treatment as well as prevention of bone loss associated with disuse, immobilization, and ovariectomy [22-24]. Similar anabolic results have been found with local delivery of prostaglandins to bone [25-27]. On a historical note, it is important to recognize that the early *in vitro *studies of prostaglandins on bone focused on its resorption effects [28], while later it was realized that this resorption activity was often followed by increased bone formation that ultimately produced an increase in bone mass. It is unclear if the extensive past focus on the negative effects of PGE_2 _on tendon has limited the consideration of positive effects in tendon healing. While the elevated level of PGE_2 _seen with cyclical loading and exercise may contribute to the development of tendinopathy, our results suggests that they may play more of an adaptive role in improving tendon strength, similar to their role in bone remodeling.

Another factor which may explain how PGE_2 _may have both positive and negative effects may be in the relative expression of the cellular receptors which mediate the response to PGE_2_. It is known that the cellular response to PGE_2 _is mediated by four G-protein coupled EP receptors. In bone, it has been shown that the EP2 and EP4 receptors regulate the anabolic response to PGE_2 _and agonists to these receptors have been shown to accelerate fracture healing [29]. It is unclear if the same EP receptors are anabolic for tendon as for bone and how receptor expression may change across stages of tendon healing. Receptor-selective EP agonists may allow for the positive effects of prostaglandins on tendon healing to be more effectively harnessed.

Our study was not without limitations. Early time points of evaluation were not included in the study and these may have helped capture early but transient responses of the tendon injections. Only one dosage was used, but this was the same dosage that was used in a past study that reported significant histological changes in tendon in a rat model [6]. The method of PGE_2 _delivery, injection, may have also contributed to the tendon response. Finally, our sample size may not have provided us adequate power to detect differences in all of the evaluation measures.

Despite these limitations, our study provided the novel finding that weekly PGE_2 _injections for four weeks can cause an improvement in the biomechanical properties of tendon and these improvements can persist for 4 weeks after ceasing injections. Future studies will have to better determine the biophysical and biochemical mechanisms by which the PGE_2 _injections caused the improvement in biomechanical properties. Weekly injection of PGE_2 _alone in the rat patellar tendon model does not appear to mimic clinical tendinopathy, and at this stage it appears more study is needed before attempting to use a prostaglandin injection model to study treatments for human tendinopathies.

## Conclusions

Four weekly peritendinous injections of PGE_2 _to the rat patellar tendon were not found to be an effective model of clinical tendinopathy. In contrast, improved structural properties of the patellar tendon were found after PGE_2 _injection. While PGE_2 _has been thought to have a contributory role in the development of tendinopathy and anti-inflammatory medications remain a common treatment, our results suggest a positive role of PGE_2 _in tendon remodeling in some circumstances.

## Competing interests

The authors declare that they have no competing interests.

## Authors' contributions

STF assisted with study conception, conducted animal procedures, performed collagen content evaluation, drafted manuscript. HMA: conducted animal procedures, performed collagen content and mechanical testing, critically reviewed manuscript. JL: performed mechanical testing and histology, critically reviewed manuscript. LED helped conceive study, participated in its design and coordination, and critically reviewed the manuscript. PSW helped conceive the study, participated in its design and coordination, conducted statistical analysis, and helped draft the manuscript. All authors have read and approved the final manuscript.
